# Supplementary material for: Ecological divergence of sibling allopolyploid marsh orchids is associated with species specific plasticity and distinct fungal communities
Source: Plant J. 2025 Feb 19;121(4):e70001. doi: 10.1111/tpj.70001 (PMC11836771; doi:10.1111/tpj.70001)
Supplement: Supplementary file 1 — Figure S1. PCA plot based on genotypic variability expressed as genotype likelihoods in Dactylorhiza majalis and D. traunsteineri in the two localities tested (Kitzbühel and St. Ulrich am Pillersee). Figure S2. Clades identified to be explanatory of the difference between fungal community profiles in D. majalis and D. traunsteineri environments using linear discriminant analysis effect size (LEFSe). Figure S3. Boxplot showing FPKM normalised read counts mapping to representative ITS sequences of five selected fungal taxa reported to form mycorrhizal associations with orchid species. Figure S4. Heatmap of FPKM normalised ITS read counts across all samples of Dactylorhiza majalis and D. traunsteineri. [file TPJ-121-0-s001.pdf]

Supporting figures

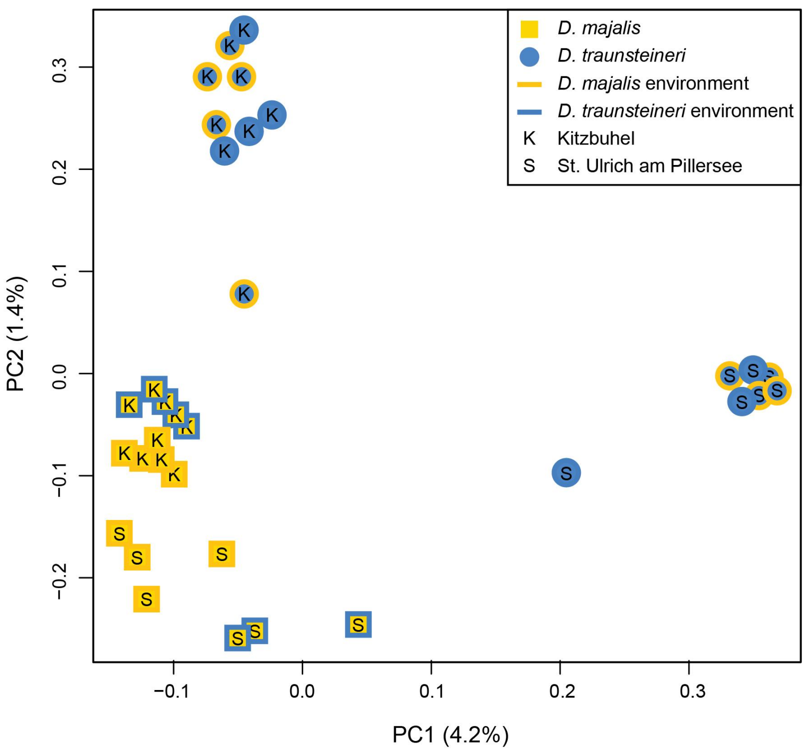

Figure S1. PCA plot based on genotypic variability expressed as genotype likelihoods in *Dactylorhiza majalis* and *D. traunsteineri* in the two localities tested (Kitzbühel and St. Ulrich am Pillersee). Symbols represent replicates of *D. majalis* (squares) and *D. traunsteineri* (circles) in native and transplanted habitats (according to the legend).

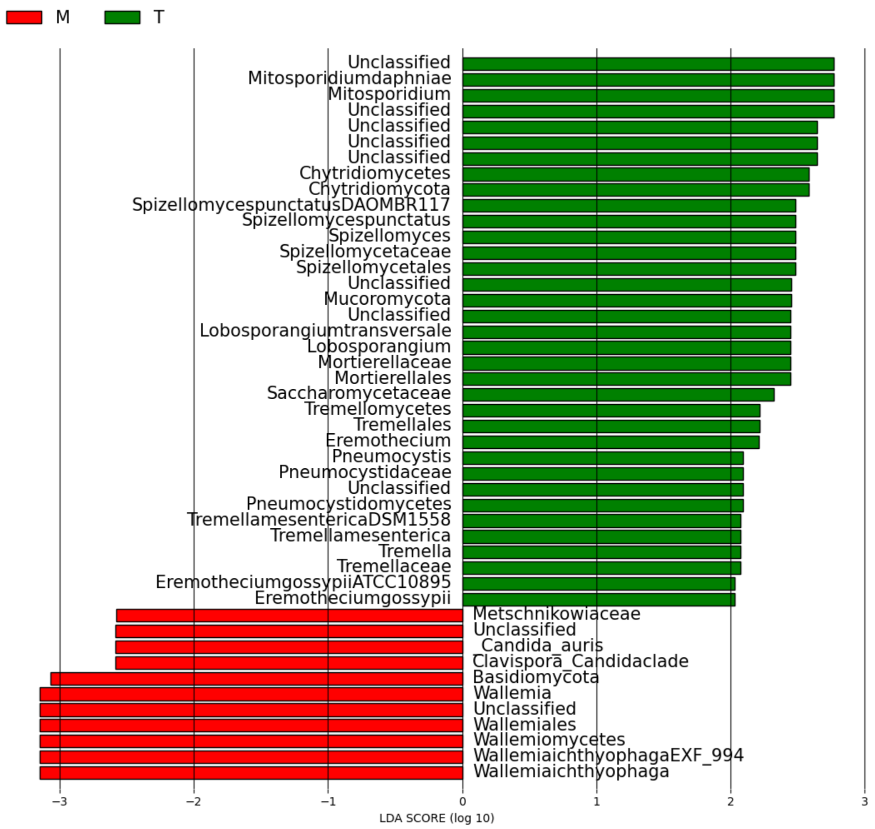

Figure S2. Clades identified to be explanatory of the difference between fungal community profiles in *D. majalis* and *D. traunsteineri* environments using linear discriminant analysis effect size (LEFSe). Only clades with a minimum log(LDA) score of 3 and a Kruskal-Wallis p-value < 0.05 are shown.

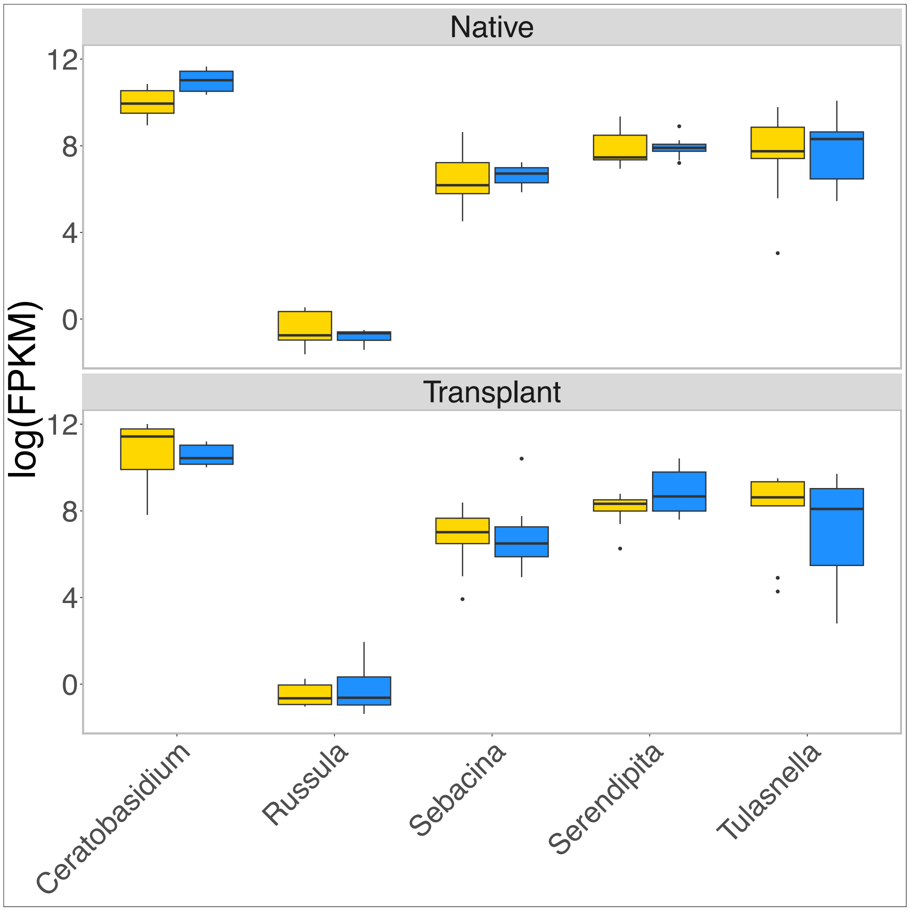

Figure S3. Boxplot showing FPKM normalised read counts mapping to representative ITS sequences of five selected fungal taxa reported to form mycorrhizal associations with orchid species. FPKM normalised read counts are shown on a log scale.

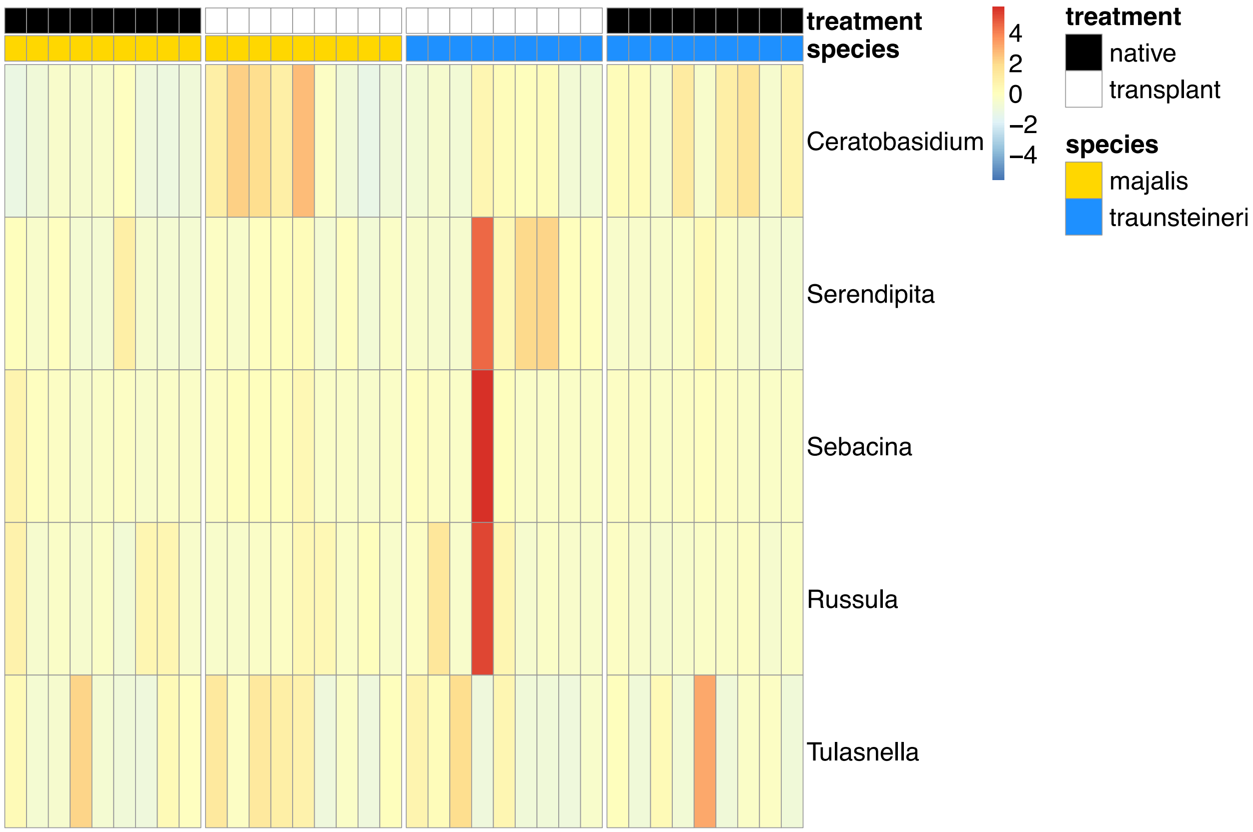

Figure S4. Heatmap of FPKM normalised ITS read counts across all samples of *Dactylorhiza majalis* and *D. traunsteineri*. Treatment of samples (native or transplant) is shown in black and white on the top annotation bar, and species is shown in blue and yellow on the bottom annotation bar. FPKM normalised counts are scaled prior to visualisation.
